# Magnetic Adsorbent Fe_3_O_4_/ZnO/LC for the Removal of Tetracycline and Congo Red from Aqueous Solution

**DOI:** 10.3390/molecules28186499

**Published:** 2023-09-07

**Authors:** Anjiu Zhao, Qi Tang, Yuanlong Chen, Chongpeng Qiu, Xingyan Huang

**Affiliations:** College of Forestry, Sichuan Agricultural University, Chengdu 611130, China; anjiu_zhao@sicau.edu.cn (A.Z.); tq15730744986@mail.ustc.edu.cn (Q.T.); chenyuanlong2017@outlook.com (Y.C.)

**Keywords:** adsorption, magnetic, zinc oxide, tetracycline, congo red

## Abstract

Zeolitic imidazolate frameworks (ZIFs) can be used as an adsorbent to efficiently adsorb organic pollutants. However, ZIF nanoparticles are easy to form aggregates, hampering the effective and practical application in practical adsorption. In this study, the ZIF-8 was successfully loaded onto lignocellulose (LC) to further produce ZnO/LC by in situ growth method and hydrothermal treatment, and then Fe_3_O_4_ nanoparticles (Fe_3_O_4_ NPs) were loaded onto ZnO/LC to prepare magnetic Fe_3_O_4_/ZnO/LC adsorbent for removing tetracycline (TC) and congo red (CR) pollutants from aqueous solution. The adsorption properties of the adsorbent were systematically analyzed for different conditions, such as adsorbent dosage, solution pH, contact time, temperature and initial concentration. The experimental data were fitted using adsorption kinetic and isotherm models. The results showed that the pseudo-second-order model and Sips model were well fitted to the adsorption kinetic and adsorption isotherm, respectively. The adsorption capacities of TC and CR reached the maximum value of 383.4 mg/g and 409.1 mg/g in experimental conditions. The mechanism of the removal mainly includes electrostatic interaction, hydrogen bonding and π-π stacking. This novel adsorbent could be rapidly separated from the aqueous solution, suggesting its high potential to remove pollutants in wastewater.

## 1. Introduction

With the rapid development of the global economy, many organic (such as dyes, pharmaceutical drugs, surfactants, etc.) and inorganic (e.g., heavy metal ions and eutrophication ions) pollutants have been released into the global water environment without scientific treatment [1,2], which have great threats to ecosystem biodiversity and human health [3]. Tetracycline (TC), a common antibiotic group used in animal husbandry, can cause tanhe increase in resistance to the microorganism, and then become a big public health crisis [4]. Meanwhile, congo red (CR) has been widely used as a dye in industries, for example, dyeing, medicine, paper, printing, etc. [5,6,7]. However, due to the unregulated and excessive use of chemicals, large amounts of toxic waste and colored dyes have been discharged into nature, causing water, air and soil pollution, resulting in a significant increase in human genetic mutations, cancers, tumors and other diseases. Dyes also have a considerable adverse effect on fisheries and livestock [8,9]. Dyes have high thermal and hydrolytic stability, which makes them extremely difficult to biodegrade in nature, thereby it is difficult to remove them from industrial wastewater [10,11]. Hence, the removal of TC and CR from the aqueous solution is a meaningful act to ensure the sustainable development of human beings.

To date, various techniques, including biological treatment [12,13], membrane separation [14,15], photocatalysis [16,17], electrochemical degradation [18,19] and adsorption [20,21], have been exploited to remove TC and CR from wastewater. Among these methods, adsorption is considered the most promising treatment method owing to its facile and economical properties [20,22]. Various adsorbents, such as natural biopolymers, biochar, zeolite, activated carbon, etc., have been applied in the purification of wastewater [23,24]. Compared with commonly used adsorbents, lignocellulose (LC) is an easy-access, biodegradable, and low-cost adsorbent for removing organic pollutants [25]. However, due to the poor adsorption capacity of raw LC, it is necessary to improve its adsorption ability.

Nowadays, metal-oxide-based nanomaterials are considered promising materials for water treatment [26]. Among these metal-oxides, zinc oxide (ZnO) is well known as one of the most versatile nanostructured semiconductors with its unique electrical and optical properties [27]. ZnO nanomaterials have been widely applied in the fields of photocatalyst [28], solar cells [29] and sensors [30], but have not been usually used as adsorbent for the remediation of wastewater. However, due to its ease of availability, high specific surface area and positive surface charge at circumneutral pH, ZnO nanomaterials may have a great potential for the adsorption of organic pollutants [26]. Hence, the adsorption capacity of LC can be greatly improved by the modification of ZnO nanomaterials. The well-designed ZnO/LC has the potential to be applied for efficient adsorption of organic pollutants from wastewater. However, it is still a problem for the poor loading of ZnO onto LC under low-temperature reaction conditions.

Zeolitic imidazolate framework-8 (ZIF-8) is a representative member of metal-organic frameworks constructed by tetrahedral coordination of zinc cations to nitrogen in 2-methylimidazole (2-MIM) linkers. ZIF-8 has been applied as an adsorbent for dyes [31], heavy metal ions [32] and pharmaceutical remediation [21]. To increase the load of ZIF-8 on lignocellulose, the ZIF-8 can be efficiently loaded onto lignocellulose by an in situ growth method [33]. The surface of lignocellulose has a large number of groups, i.e., hydroxyl groups, which can react with the organic ligand 2-methylimidazole to form hydrogen bonds, and then the coordinated 2-methylimidazole binds to Zn^2+^ and grows on the surface to form crystals. However, several recent studies show that ZIF-8 is not stable in aqueous and extremely acidic solutions [34]. ZIF-8 has been used as a sacrificial precursor to obtain ZnO with excellent stability, large specific surface area and nano/micro-structures by hydrothermal treatment at 80 °C [35]. Therefore, the ZnO/LC composite can be prepared by hydrothermal treatment of the in situ growth of ZIF-8 on LC.

It is difficult to recycle the powdered adsorbents in practical applications, which need to be separated by filtration or centrifugation. It may increase the complexity and cost of reusability [36]. Fe_3_O_4_ nanoparticles (Fe_3_O_4_ NPs) have the advantages of stable chemical properties, high adsorption capacity, large specific surface area and easy magnetic separation [37]. Therefore, loading Fe_3_O_4_ NPs onto the ZnO/LC composites can be an efficient strategy to avoid these defects [38].

We herein report a simple method for the preparation of Fe_3_O_4_/ZnO/LC from LC, ZIF-8 and Fe_3_O_4_ NPs for the application of wastewater purification. The Fe_3_O_4_/ZnO/LC was characterized by FTIR, XRD, VSM, BET, XPS and SEM-EDS, and the adsorption behavior of TC and CR have been systematically investigated. The kinetic and isothermal studies were performed under different experimental conditions. Furthermore, the adsorption mechanism for TC and CR by Fe_3_O_4_/ZnO/LC was proposed in this work. The adsorbent prepared in this study has the advantages of a simple preparation process, with high adsorption efficiency, strong magnetism and easy recovery, and it has the potential for large-scale industrial application.

## 2. Results and Discussion

### 2.1. Characterization of Fe_3_O_4_/ZnO/LC

**Figure 1:**
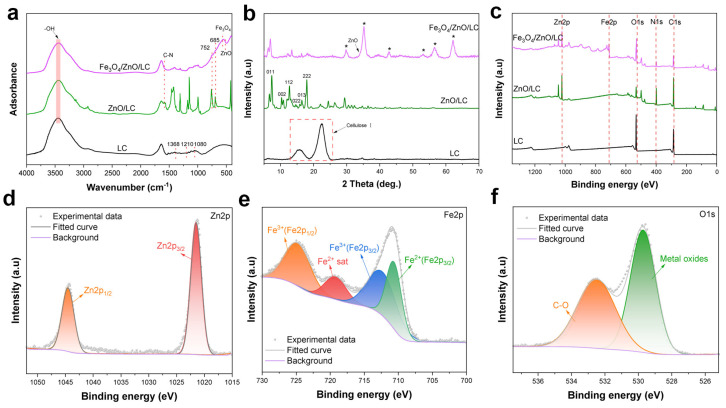
FTIR spectra (**a**); XRD patterns (**b**); and XPS full spectrum (**c**) of the LC, ZIF-8/LC and Fe_3_O_4_/ZnO/LC; (**d**–**f**) Zn 2p, Fe 2p and O 1s XPS spectrum of Fe_3_O_4_/ZnO/LC.

FTIR spectra were measured to analyze the functional groups of the LC, ZIF-8/LC and Fe_3_O_4_/ZnO/LC. As shown in Figure 1a, the peaks at 3440, 1368, 1210 and 1080 cm^−1^ were assigned to -OH stretching, the phenolic band, aromatic C-O stretch and secondary aliphatic alcohol stretch, respectively [3]. It indicated that the LC was successfully prepared compared with LC, and many new peaks appeared in ZIF-8/LC. For example, the characteristic peaks at 1579, 752 and 685 cm^−1^ were attributed to the C-N stretching and the entire imidazole ring stretching of ZIF-8 [39]. For Fe_3_O_4_/ZnO/LC, the band at 587 cm^−1^ corresponded to the adsorption peak of Fe-O, probably exhibiting that the ferroferric oxide nanoparticles were loaded onto the Fe_3_O_4_/ZnO/LC [4]. The band at 496 cm^−1^ was attributed to the bending vibration of Zn-O in ZnO. Meanwhile, the characteristic peaks of imidazole ring stretching of ZIF-8 were also weakened in Fe_3_O_4_/ZnO/LC. It was due to the ZnO formed from ZIF-8 after hydrothermal treatment [36]. These results demonstrated that the Fe_3_O_4_/ZnO/LC was successfully synthesized.

The LC, ZIF-8/LC and Fe_3_O_4_/ZnO/LC were characterized via XRD to monitor the changes in their crystalline structure. As revealed in Figure 1b, the characteristic peaks of 2θ = 14.6°, 16.7° and 22.2° were ascribed to (101), (101) and (002) planes in typical native cellulose I structure of LC [40]. After the in situ growth of ZIF-8 onto LC, the cellulose I characteristic diffraction peaks were weakened in ZIF-8/LC. Meanwhile, the diffraction peaks of ZIF-8/LC at 2θ = 7.4°, 10.2°, 12.8°, 14.7°, 16.5° and 18.1° were assigned to (011), (002), (112), (022), (013) and (222) planes of ZIF-8, respectively [32]. These results indicated the triumphant in situ growth of ZIF-8 onto LC. The XRD pattern of Fe_3_O_4_/ZnO/LC showed the characteristic diffraction peaks at 2θ = 30.1°, 35.5°, 43.2°, 53.3°, 57.0° and 62.7°, which were corresponded to (220), (311), (400), (422), (511) and (440) planes, respectively, indicating that the inverse spinel-type Fe_3_O_4_ was loaded onto ZIF-8/LC [4]. The characteristic diffraction peaks of ZIF-8 almost disappeared in Fe_3_O_4_/ZnO/LC. Meanwhile, the characteristic diffraction peak at 2θ = 34.4° belonged to ZnO. This was because the ZIF-8 was almost destroyed to form ZnO [36]. The surface chemical compositions of the LC, ZIF-8/LC and Fe_3_O_4_/ZnO/LC samples were evaluated by XPS. As shown in Figure 1c, the major peaks in the LC were C1s and O1s. In contrast to the spectrum of LC, a Zn2p peak appeared in ZIF-8/LC, which was assigned to the Zn element of ZIF-8. Compared with the spectrum of ZIF-8/LC, a Fe2p peak appeared in Fe_3_O_4_/ZnO/LC, while the peak of Zn2p was weakened in Fe_3_O_4_/ZnO/LC. This was probably due to the Fe element of Fe_3_O_4_ and the formation of ZnO [41]. In Figure 1d, the binding energies of 1021.70 and 1044.75 eV were assigned to Zn 2p_3/2_ and Zn 2p_1/2_, respectively. In the spectrum of Fe 2p (Figure 1e), the obvious binding energies of 710.6 and 712.3 eV were assigned to Fe^2+^ and Fe^3+^ (Fe 2p_3/2_), respectively. A satellite peak at 718.9 eV may be attributed to the presence of Fe^2+^ [4], and the highest bonding energy peak at 724.7 eV was ascribed to the Fe^3+^ (Fe 2p_1/2_) of Fe_3_O_4_ [21]. The results indicated the existence of Fe_3_O_4_, which was in agreement with the FTIR and XRD results. The O1s spectrum of Fe_3_O_4_/ZnO/LC (Figure 1f) was divided into two peaks at 529.8 and 531.7 eV for metal-oxides (ZnO and Fe_3_O_4_) and C-O groups, respectively [42]. These results indicated that the ZnO and Fe_3_O_4_ were successfully loaded onto the LC.

**Figure 2:**
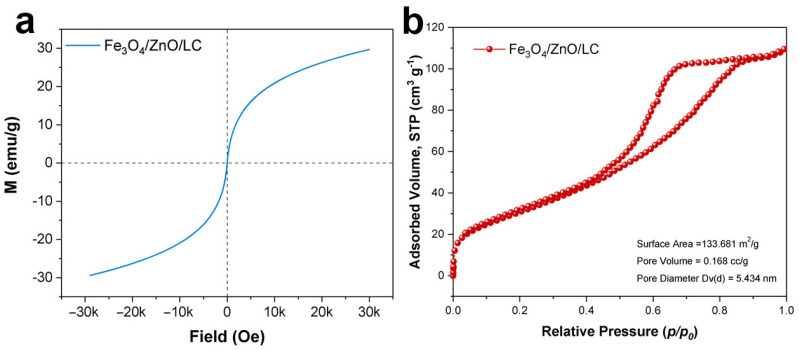
VSM of Fe_3_O_4_/ZnO/LC (**a**); N_2_ adsorption–desorption isotherms of Fe_3_O_4_/ZnO/LC (**b**).

The magnetic hysteresis curve of Fe_3_O_4_/ZnO/LC was evaluated, as noticed in Figure 2a. The saturation magnetization of Fe_3_O_4_/ZnO/LC could reach 23.7 emu/g, which was much higher than that of β-CD@MRHC (17.4 emu/g) [43]. Thus, the superparamagnetic characteristic Fe_3_O_4_/ZnO/LC was demonstrated to be effective in separating the adsorbent from water under the function of an outer magnetic field [4]. It was evidenced from Figure 2b that the N_2_ adsorption/desorption isotherms of Fe_3_O_4_/ZnO/LC belonged to type-IV adsorption/desorption isotherms, which were typical for mesoporous materials [26]. Meanwhile, the BET results also indicated that the Fe_3_O_4_/ZnO/LC adsorbent had numerous small size pores. This pore structure was beneficial for providing more accessible adsorption sites for TC and CR uptake. It suggested that the Fe_3_O_4_/ZnO/LC had good potential for practical application.

**Figure 3:**
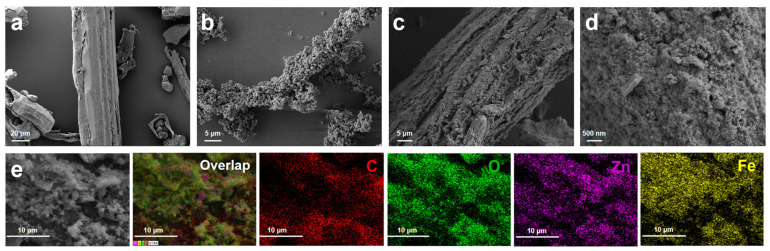
SEM images of (**a**) LC, (**b**) ZIF-8/LC and (**c**,**d**) Fe_3_O_4_/ZnO/LC; EDS mapping images of C, O, Zn and Fe on the Fe_3_O_4_/ZnO/LC. (**e**) is the amplification image of (**d**).

The surface morphologies of LC, ZIF-8/LC and Fe_3_O_4_/ZnO/LC were evaluated. As shown in Figure 3a, typical fiber structures were clearly observed from LC, ZIF-8/LC and Fe_3_O_4_/ZnO/LC. In addition, the surface of LC was covered by ZIF-8, which proved the successful in situ growth of ZIF-8 onto LC (Figure 3b). Moreover, there were some nanospheres formed on the surface and channel of Fe_3_O_4_/ZnO/LC (Figure 3c,d), suggesting successful loading of Fe_3_O_4_ nanoparticles. Meanwhile, some rod-shaped nanoparticles appeared on the surface of Fe_3_O_4_/ZnO/LC. It was probably attributed to ZnO particles transformed by ZIF-8 after hydrothermal treatment [21]. The element (C, O, Zn and Fe) composition and distribution on Fe_3_O_4_/ZnO/LC were investigated by EDS elemental mapping. Furthermore, the element mapping images of Fe_3_O_4_/ZnO/LC showed that C, O, Zn and Fe were evenly distributed on the Fe_3_O_4_/ZnO/LC surface (Figure 3e).

### 2.2. Adsorption Study

#### 2.2.1. Comparison of Adsorption Properties of LC, ZnO/LC and Fe_3_O_4_/ZnO/LC

As shown in Table 1, compared with LC, the adsorption performance of ZnO/LC for TC and CR improved slightly. When Fe_3_O_4_ was introduced, the adsorption performance of the adsorbent has been remarkably improved. It was due to the increase in the available adsorption sites in ZnO/LC/Fe_3_O_4_. It was suggested that Fe_3_O_4_ had a promoting effect on the adsorption performance of the adsorbent.

#### 2.2.2. Effect of Temperature on the TC and CR Adsorption

**Figure 4:**
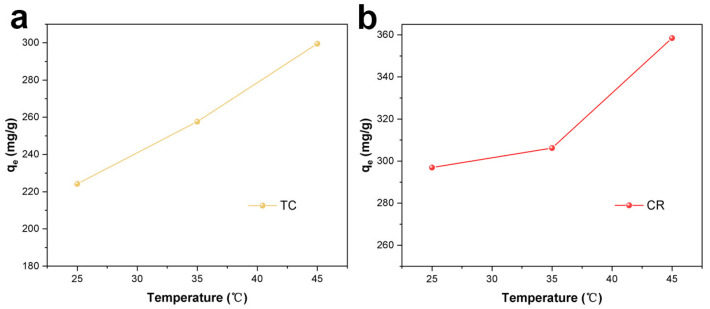
The effect of solution temperature on the TC (**a**) and CR (**b**) adsorption.

Figure 4 shows the change of adsorption performance of the adsorbent from 25 °C to 45 °C. As the temperature increased, the adsorption performance of Fe_3_O_4_/ZnO/LC had been slightly improved. However, as compared with the performance at room temperature, the adsorption capacity caused by the increase in temperature was not remarkable. Therefore, room temperature (25 °C) was used to carry out the subsequent experiments.

#### 2.2.3. Effect of Adsorbent Dosage on the TC and CR Adsorption

**Figure 5:**
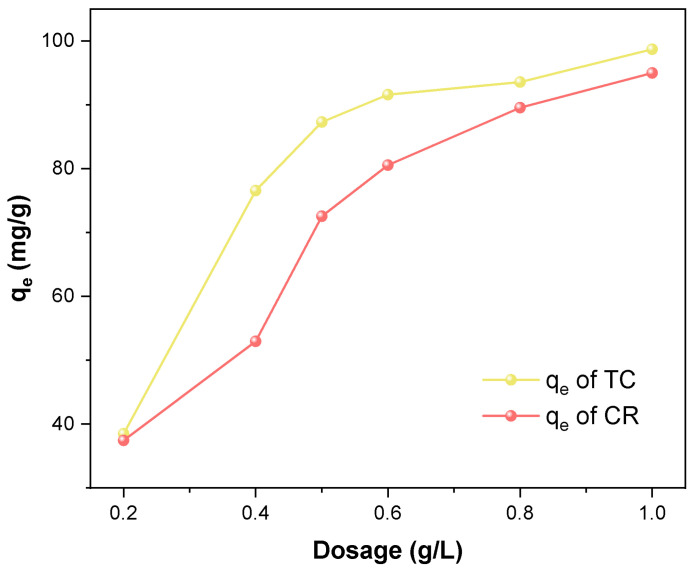
The effect of adsorbent dosage on T and CR adsorption process.

The effect of the Fe_3_O_4_/ZnO/LC dosage on the adsorption is shown in Figure 5. The adsorption capacity of Fe_3_O_4_/ZnO/LC increased with the increase in adsorbent dosage from 0.2 to 1.0 g/L. However, when the adsorbent dose exceeded 0.5 g/L, the increase rate of adsorption capacity was reduced, which indicated that the adsorption capacity of Fe_3_O_4_/ZnO/LC tended to be saturated. This phenomenon could be ascribed to the aggregation and overlap of adsorption sites caused by the excessive adsorbents [44]. Therefore, 0.5 g/L Fe_3_O_4_/ZnO/LC was selected as the optimal adsorbent dosage in this study.

#### 2.2.4. Effect of Solution pH on the TC and CR Adsorption

**Figure 6:**
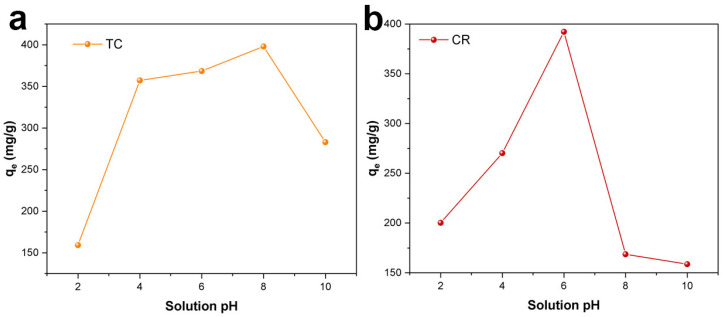
The effect of solution pH on TC (**a**) and CR (**b**) adsorption process.

The solution pH of TC and CR are the most important factors in the adsorption process. The existence state of TC and CR molecules and the surface charge of the Fe_3_O_4_/ZnO/LC can be influenced by solution pH [45]. Therefore, the effect of solution pH on TC and CR adsorption was investigated in this work. As shown in Figure 6a, the adsorption capacity of TC increased first and then decreased as solution pH > 8. When the solution pH was lower than 3.5, the low TC adsorption capacity was ascribed to the electrostatic repulsion between the positive charge of TC (TCH_3_^+^) and Fe_3_O_4_/ZnO/LC (protonation of -OH) [46]. The increase in TC adsorption capacity was attributed to the weakened electrostatic repulsion for the zwitterion TCH_2_^0^ formed by deprotonation of TC within solution pH values of 3.5–7.5 [45]. When the solution pH was higher than 7.5, there was an electrostatic repulsion between the negative charge of TC (TCH- or TC2-) and Fe_3_O_4_/ZnO/LC, resulting in a decrease in TC adsorption capacity [47].

As shown in Figure 6b, the adsorption capacity of Fe_3_O_4_/ZnO/LC for CR increased from 100.2 to 196.1 mg/g, and that for CR was increased by 200.1 to 376.8 mg/g with increasing solution pH from 2 to 6 (Figure 6b). This might be attributed to the electrostatic attraction between amine group protonation (-NH_3_^+^) of Fe_3_O_4_/ZnO/LC and sulfonated groups (-SO^3−^) of CR in acid solution [48]. The ionic interaction between CR and -NH^3+^ in Fe_3_O_4_/ZnO/LC was competed by the huge numbers of H_3_O^+^, resulting in the reduction in CR adsorption capacity [49]. As the pH increased from 6 to 10, the adsorption capacity of Fe_3_O_4_/ZnO/LC on CR significantly decreased, which may be due to the increase in negative charge on the surface of Fe_3_O_4_/ZnO/LC, resulting in its weakened electrostatic attraction with CR.

#### 2.2.5. Adsorption Kinetics

Adsorption kinetic is an index to analyze the process of adsorption experiments, adsorbent properties and adsorption mechanism by studying the rate of material transfer. In this study, three models were explored: the pseudo-first-order model (Equation (1)), the pseudo-second-order model (Equation (2)) and the Weber–Morris intraparticle diffusion model (Equation (3)) [50]. The model was fitted based on known data *t* (min) and *q_e_* (mg·g^−1^) to analyze the adsorption mechanism.

(1) $$q_{t}=q_{e}\times \left(1-e^{-k_{1}t}\right)$$

(2) $$q_{t}=\frac{q_{e}^{2}k_{2}t}{1+q_{e}k_{2}t}$$

(3) $$q_{t}=k_{d}t^{0.5}+C$$

where *t* is contact time (h); *q_e_* is the adsorbed concentration of TC and CR (mg/g) at equilibrium; *q_t_* is the equilibrium concentration of TC and CR (mg/g) at time *t*; *k*_1_ and *k*_2_ are the rate constants (g/mg·h); *k_d_* is the intraparticle diffusion rate constant (mg/g·min); and *C* is the thickness of the boundary layer at different stages of the intraparticle diffusion model.

**Figure 7:**
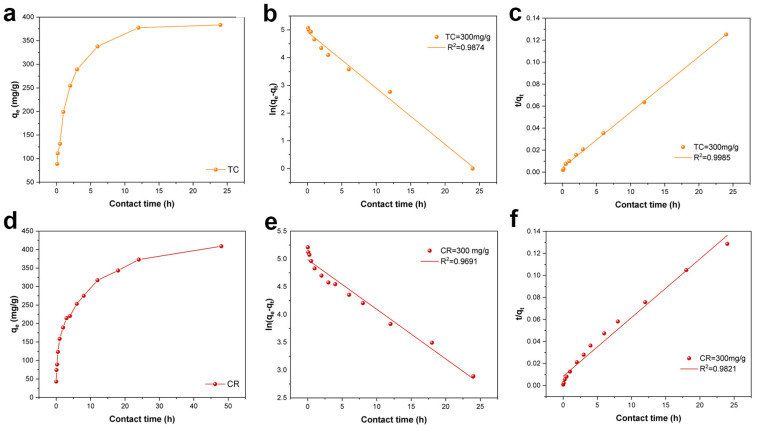
The effect of contact time on the adsorption of (**a**) TC and (**d**) CR by Fe_3_O_4_/ZnO/LC; Linear plots of pseudo 1st order model for (**b**) TC and (**e**) CR uptake; Linear plots of pseudo 2nd order model for (**c**) TC and (**f**) CR uptake.

**Figure 8:**
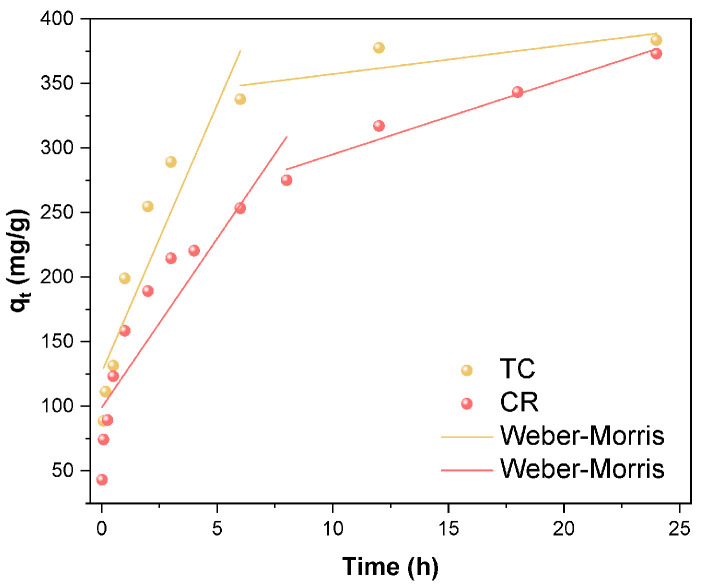
The intraparticle diffusion of TC and CR by Fe_3_O_4_/ZnO/LC.

As shown in Figure 7a, the amount of TC and CR adsorbed on the Fe_3_O_4_/ZnO/LC both increased rapidly with a prolonged contact time and then tended to be equilibrium. It was ascribed to the adsorption sites of Fe_3_O_4_/ZnO/LC decreased with contact time [33]. The fitting results and kinetic parameters are shown in Figure 7b–f and Table 2. By comparing the determination coefficients (*R*^2^) of all models, it could be concluded that the adsorption of CR onto Fe_3_O_4_/ZnO/LC was better fitted to the pseudo-second-order model. It indicated that the adsorption process of TC and CR on Fe_3_O_4_/ZnO/LC involved a chemical process of electron sharing and electron transfer [51,52]. Therefore, it could be speculated that the adsorption performances of Fe_3_O_4_/ZnO/LC for TC and CR were mainly controlled by chemical adsorption. According to the internal diffusion model (Figure 8), the adsorption process could be divided into two stages: first, TC and CR were absorbed by the fast adsorption part of the adsorbent, which combined the effects of surface contact mass transfer and internal diffusion; the second stage, TC and CR were slowly diffused into the pores of the adsorbent until adsorption saturation was reached. The linear fitting curve did not pass through the origin, confirming that the internal diffusion mechanism of the particles was not the rate-limiting step of the TC and CR adsorption process and that there were other adsorption forces such as surface distribution [53].

#### 2.2.6. Adsorption Isotherms

Adsorption isotherm models are usually used to characterize the adsorption function and adsorption mechanism of adsorbents, which reflect the relationship between adsorbate and adsorbent mass. The Langmuir (Equation (4)), Freundlich (Equation (5)), Sips (Equation (6)) and Weber–Morris (Equation (7)) isotherm models were studied to evaluate the adsorption performance of Fe_3_O_4_/ZnO/LC to TC and CR [54].

(4) $$q_{e}=\frac{K_{L}q_{m}C_{e}}{1+K_{F}C_{e}}$$

(5) $$q_{e}=K_{F}C_{e}^{\frac{1}{n}}$$

(6) $$q_{e}=\frac{q_{m}K_{S}C_{e}^{1/n}}{1+K_{S}C_{e}^{1/n}}$$

(7) $$q_{e}=B_{T}\mathrm{Ln}K_{T}+B_{T}LnC_{e}$$

where *q_e_* is the equilibrium dye concentration on the adsorbent (mg/g); *C_e_* is the concentration of dye at equilibrium (mg/L); *q_m_* is the maximum adsorption capacity (mg/g); *n* is the exponent (dimensionless); B_T_ is a constant related to Temkin adsorption heat (J mol^−1^); and *K_F_*, *K_L_*, *K_S_*, and *K_T_* represent Freundlich, Langmuir, Sips and Weber–Morris adsorption constants [55].

One of the essential characteristics of the Langmuir isotherm can be expressed by a dimensionless constant, separation factor, *R_L_* is defined as Equation (8) [56]:

(8) $$R_{L}=\frac{1}{1+{Κ}_{L}C_{0}}$$

where *R_L_* is a dimensionless equilibrium parameter or the separation factor and *C*_0_ is the initial dye concentration of adsorbate solution (mg/L). The value of *R_L_* denotes the adsorption nature to be unfavorable (*R_L_* > 1), favorable (0 < *R_L_* < 1), irreversible (*R_L_* = 0) or linear (*R_L_* = 1).

**Figure 9:**
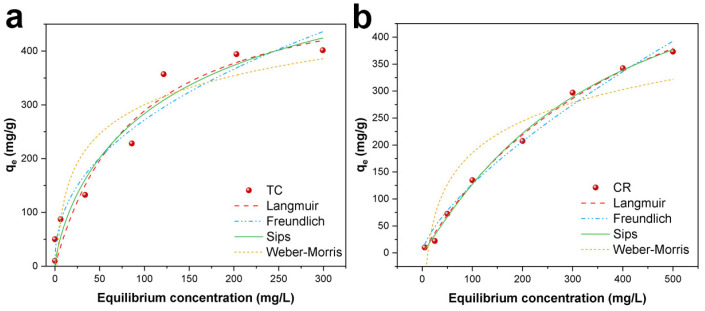
Isotherm plots for the adsorption of (**a**) TC and (**b**) CR by Fe_3_O_4_/ZnO/LC.

The fitting results and parameters of these four adsorption isotherms have been summarized in Figure 9 and Table 3. The correlation coefficient of the three models indicated that the adsorption of TC and CR on the Fe_3_O_4_/ZnO/LC was better fitted with Sips isotherm. It suggested that the adsorption of TC and CR onto the Fe_3_O_4_/ZnO/LC was a combined model: diffuse (at low concentration) and monomolecular (at high concentration) [33]. Meanwhile, the calculated *R_L_* from the Langmuir model was between 0 and 1, demonstrating that the adsorptions of TC and CR onto the Fe_3_O_4_/ZnO/LC were favorable. The adsorption amounts of TC and CR calculated from the Sips isotherm model were 678.6 and 548.0 mg/g (Table 3), respectively, which were higher than most of the previously reported works (Table 4).

### 2.3. Adsorption Mechanism

**Figure 10:**
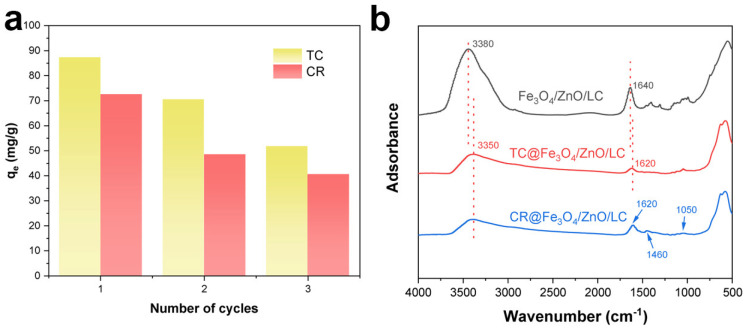
Three cycles of TC and CR adsorption and desorption (**a**); FTIR spectra of Fe_3_O_4_/ZnO/LC before and after TC and CR adsorption (**b**).

The Fe_3_O_4_/ZnO/LC had a large specific surface area and a rich pore structure to facilitate the adsorption of TC and CR molecules. Figure 10a illustrates the removal efficiency during the adsorption of TC and CR after three cycles. The removal rate remained above 50% after three cycles. These results indicated that the Fe_3_O_4_/ZnO/LC had high stability and excellent reusability, which was a promising adsorbent for the TC and CR removal. The adsorption performances of TC and CR were related to the change in the initial solution’s pH. It was ascribed to the electrostatic attraction between the adsorbent and the pollutant. In order to further clarify the adsorption mechanism of TC and CR onto Fe_3_O_4_/ZnO/LC, the functional groups on the surface of Fe_3_O_4_/ZnO/LC before and after adsorption of TC and CR were detected by FTIR (Figure 10b). It could be seen that the IR spectra after the adsorption spectrum were different from that of the original Fe_3_O_4_/ZnO/LC, which was certainly due to the adsorption of TC and CR molecules. The C-H band at 2925–2964 cm^−1^ disappeared, which could be due to the extensive removal of hydrogen during the adsorption process. It could be seen that the peak originally at 3380 cm^−1^ was shifted to 3350 cm^−1^, which was due to the overlapping of the stretching vibration of the O-H and N-H, as well as the intermolecular hydrogen bonding with TC and CR molecules [62]. After the adsorption of TC and CR, the peak at 1640 cm^−1^ shifted to 1620 cm^−1^ after adsorption. This could be attributed to the π-π stacking interaction. After the adsorption of CR, new peaks at 1460 and 1050 cm^−1^ were observed due to the N=N stretching vibration and SO^3−^ group, respectively [63]. Therefore, the mechanism of the TC adsorption on Fe_3_O_4_/ZnO/LC mainly includes electrostatic interaction, hydrogen bonding and π-π stacking [64].

## 3. Materials and Methods

### 3.1. Materials

The bamboo powder was ground from waste moso bamboo. Zinc acetate dihydrate (98%, C_4_H_6_O_4_Zn·2H_2_O), 2-methylimidazole (≥99.5%), iron chloride hexahydrate (99%, FeCl_3_·6H_2_O), iron chloride tetrahydrate (98%, FeCl_2_·4H_2_O), tetracycline (TC, 96% in purity) and choline chloride (C_4_H_14_ClNO, 98%) were purchased from Shanghai Macklin Biochemical Co., Ltd. (Shanghai, China). Oxalic acid dihydrate, sodium hydroxide (NaOH), and methanol were provided by Kelong Chemical Reagent Co., Ltd. (Chengdu, China). All the chemicals were used directly without further purification. Congo red (CR) was obtained from Sinopharm Chemical Regent Co., Ltd. (Shanghai, China).

### 3.2. Synthesis of Fe_3_O_4_/ZnO/LC

**Figure 11:**
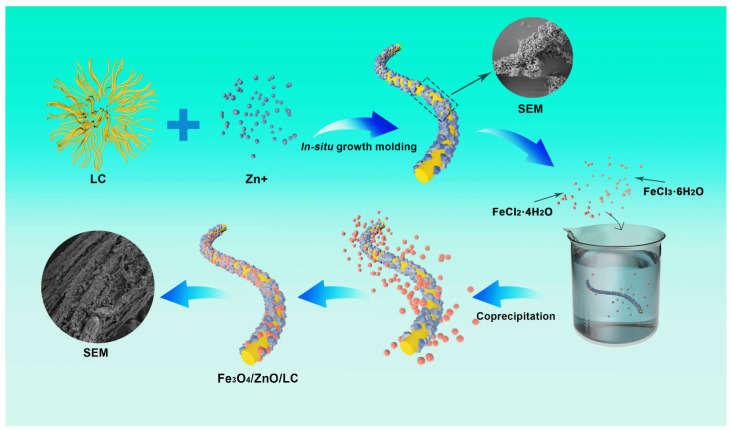
Synthetic procedure of Fe_3_O_4_/ZnO/LC.

Fe_3_O_4_/ZnO/LC was fabricated according to the following steps (Figure 11). In total, 0.5 g LC was dispersed in 50 mL deionized water and sonicated for 10 min to form LC suspension. The LC suspension was mixed with 1.5 g zinc acetate dihydrate for 6 h. After chelation, the 2-methylimidazole solution (3 g in 50 mL of deionized water) was then poured into the mixture and stirred for 10 min at 300 rpm. After the mixture was left for 5 h at room temperature, the precipitate was centrifuged with methanol several times as a white solid. Thereafter, the white solid was dried at 60 °C for 12 h to obtain ZIF-8/LC. The Fe_3_O_4_/ZnO/LC was prepared by a chemical co-precipitation method. In total, 1.4 g FeCl_3_·6H_2_O and 0.7 g FeCl_2_·4H_2_O were dissolved in a beaker with 450 mL deionized water and hydrothermally treated at 70 °C to dissolve them. In total, 1.625 g ZIF-8/LC was added to the solution. Then, 5M NaOH (50 mL) was added dropwise into the solution. The solution was stirred for 1 h and matured for 2 h. After removing the supernatant, the black solid was washed with deionized water to neutral pH and freeze-dried for 24 h to obtain Fe_3_O_4_/ZnO/LC.

### 3.3. Characterization

The surface morphology and composition of Fe_3_O_4_/ZnO/LC were analyzed via a scanning electron microscope (SEM, ZEISS Gemini 300, Shanghai, China) equipped with an energy dispersive spectrometer (EDS, OXFORD Xplore, Shanghai, China). The surface functional groups of Fe_3_O_4_/ZnO/LC were characterized by Fourier transform infrared spectra (FTIR, Nicolet-6700, Thermo Fisher Scientific, Waltham, MA, USA). The Fe_3_O_4_/ZnO/LC superficial chemistry properties were investigated through the X-ray photoelectron spectroscope (XPS, Thermo Fisher Scientific, Waltham, MA, USA). The crystallographic structure and phase composition characteristics of Fe_3_O_4_/ZnO/LC were analyzed by X-ray diffractometer (XRD, Rigaku SmartLab, Osaka, Japan) with Cu-Kα radiation within the 2θ range of 5–80°. The saturation magnetization was evaluated by a vibrating sample magnetometer (VSM). The specific surface area and pore size distribution of Fe_3_O_4_/ZnO/LC were determined by Brunauer Emmett Teller (BET) and Barret Joyner Halender (BJH) methods, respectively.

### 3.4. Adsorption Study

The adsorption experiments were conducted to determine the contact time (0–24 h), adsorbent dosage (0.2–1.0 g/L), temperature (25–45 °C), initial concentration (5–500 mg/L) and initial solution pH (2–10) effects on contaminants removal. All the experiments were conducted in the 100 mL Erlenmeyer flasks with 20 mL of contaminants solutions. The mixed solutions were shaken at 25 °C for a fixed time. After magnetic separation, the remaining solution was filtered using 0.45 μm millipore membrane filters. The concentration of residual TC and CR were measured by a UV–vis spectrophotometer (Unico, Shanghai, China) at wavelengths of 357 and 269.5 nm, respectively. The adsorption capacity (*q_t_*, mg/g) at time t was calculated as follows:

(9) $$q_{t}=\frac{(C_{0}-C_{t})\times V}{m}$$

where *C*_0_ (mg/L) is the initial dye concentration; *C_t_* (mg/L) is the final dye concentration at time t; *m* (mg) is the adsorbent mass and *V* (mL) is solution volume.

## 4. Conclusions

This study developed a facile approach to prepare Fe_3_O_4_/ZnO/LC adsorbent for application in the treatment of wastewater. This material was achieved via the in situ growth and transformation of ZIF-8 into ZnO on the LC surface by hydrothermal treatment. The successful preparations of ZnO and Fe_3_O_4_ NPs and their loading onto LC have been confirmed by SEM, FTIR, XRD and XPS analysis. The EDS analysis and elemental mapping revealed that ZnO and Fe_3_O_4_ NPs were dispersed well on the LC surface. The VSM analysis results showed an excellent magnetic performance of Fe_3_O_4_/ZnO/LC (23.7 emu/g). In addition, adsorption conditions such as initial concentration, adsorbent dosage solution pH, contact time and temperature had remarkable effects on the adsorption performance of Fe_3_O_4_/ZnO/LC. Isothermal adsorption data for TC and CR could be better fitted by the Sips isotherm model. The estimated maximum adsorption capacities were 678.6 and 548.0 mg/g for TC and CR, respectively, calculated by the Sips model at the optimized adsorption conditions. The present work provided a feasible pathway to develop a highly efficient magnetic adsorbent for wastewater treatment and environmental remediation. At the same time, the adsorbent has many advantages such as low cost, simple preparation and easy recovery. It has the possibility of large-scale industrial application, while its process for industrialization still needs to be further explored.

## Figures and Tables

**Table 1:** The adsorption properties of TC and CR by LC, ZnO/LC and Fe_3_O_4_/ZnO/LC.

Adsorbents	Adsorbates	
	TC	CR
LC	94.7 mg/g	109.6 mg/g
ZnO/LC	169.7 mg/g	166.8 mg/g
Fe_3_O_4_/ZnO/LC	224.2 mg/g	297.0 mg/g

**Table 2:** Parameters of TC and CR adsorption kinetic models by Fe_3_O_4_/ZnO/LC.

Models	Parameters	Adsorbates	
		TC	CR
Experimental	*C*_0_	300	300
	*q_e_*_,*exp*_ (mg/g)	383.41	409.12
Pseudo 1st order	*k*_1_ (g/mg·h)	0.793	0.0893
	*q_e_*_,*cal*_ (mg/g)	178.05	146.52
	*R* ^2^	0.8805	0.9691
Pseudo 2nd order	*k*_2_ (g/mg·h)	0.0062	−7.4595
	*q_e_*_,*cal*_ (mg/g)	193.66	169.65
	*R* ^2^	0.9402	0.9821
Weber–Morris intraparticle diffusion model	*k*_1_ (g/mg·h)	41.4784	26.1916
	*R* ^2^	0.8484	0.6847
	*K*_2_ (g/mg·h)	2.2391	5.8279
	*R* ^2^	0.8305	0.9630

**Table 3:** Parameters of TC and CR adsorption isotherm model by Fe_3_O_4_/ZnO/LC.

Models	Parameters	Adsorbates	
		TC	CR
Langmuir	*K_L_* (L/g)	0.0114	0.0083
	*q_m_* (mg/g)	543.5	517.94
	*R* ^2^	0.9474	0.9710
	*R_L_*	0 < *R_L_* < 1	0 < *R_L_* < 1
Freundlich	*K_F_*	37.3	17.37
	n	2.3186	1.8379
	*R* ^2^	0.9429	0.9687
Sips	*q_m_* (mg/g)	678.60	547.96
	*K_S_* ((mg/L)1/n)	0.0208	0.0093
	*n*	1.2999	1.0527
	*R* ^2^	0.9494	0.9818
Weber–Morris	*K_T_*	0.4651	0.08945
	*B_T_*	78.1612	84.6366
	*R* ^2^	0.7966	0.8651

**Table 4:** Comparison between various adsorbents used for CR and TC adsorption.

Adsorbent Raw Material	Contaminant	*q_max_*/mg·g^−1^	Reference
Chitosan hydrobeads	CR	92.6	[57]
Red mud	CR	4.1	[58]
Graphene oxide/chitosan fibres	CR	294.0	[59]
Fe_3_O_4_/ZnO/LC	CR	373.1	This work
Kaolinite	TC	47	[60]
Vermiculite	TC	36.8	[61]
Fe_3_O_4_/ZnO/LC	CR	308.7	This work

## Data Availability

Data are available as request to X.H.

## Sample Availability

Sample is available as request to X.H.
